# Heme on innate immunity and inflammation

**DOI:** 10.3389/fphar.2014.00115

**Published:** 2014-05-27

**Authors:** Fabianno F. Dutra, Marcelo T. Bozza

**Affiliations:** Laboratório de Inflamação e Imunidade, Departamento de Imunologia, Instituto de Microbiologia, Universidade Federal do Rio de JaneiroRio de Janeiro, Brazil

**Keywords:** heme, iron, hemolysis, ROS, inflammation, innate immunity, programed cell death, cytotoxicity

## Abstract

Heme is an essential molecule expressed ubiquitously all through our tissues. Heme plays major functions in cellular physiology and metabolism as the prosthetic group of diverse proteins. Once released from cells and from hemeproteins free heme causes oxidative damage and inflammation, thus acting as a prototypic damage-associated molecular pattern. In this context, free heme is a critical component of the pathological process of sterile and infectious hemolytic conditions including malaria, hemolytic anemias, ischemia-reperfusion, and hemorrhage. The plasma scavenger proteins hemopexin and albumin reduce heme toxicity and are responsible for transporting free heme to intracellular compartments where it is catabolized by heme-oxygenase enzymes. Upon hemolysis or severe cellular damage the serum capacity to scavenge heme may saturate and increase free heme to sufficient amounts to cause tissue damage in various organs. The mechanism by which heme causes reactive oxygen generation, activation of cells of the innate immune system and cell death are not fully understood. Although heme can directly promote lipid peroxidation by its iron atom, heme can also induce reactive oxygen species generation and production of inflammatory mediators through the activation of selective signaling pathways. Heme activates innate immune cells such as macrophages and neutrophils through activation of innate immune receptors. The importance of these events has been demonstrated in infectious and non-infectious diseases models. In this review, we will discuss the mechanisms behind heme-induced cytotoxicity and inflammation and the consequences of these events on different tissues and diseases.

## HEME PHYSIOLOGY

Heme consists of a tetrapyrrole ring with an iron (Fe) atom bound in the center, coordinated to the pyrrole rings (Kumar and Bandyopadhyay, 2005). Heme is present in organisms of all kingdoms being the prosthetic group of several proteins fundamental for the life of aerobic organisms (Wagener et al., 2003b). In vertebrates, heme is ubiquitously expressed and its functions are determined by the polypeptide interacting with it (Dawson, 1988). The capacity of the chelated Fe to undergo a reversible change in the oxidation state renders heme a versatile biological catalyst acting in oxidative reactions, electron transfer processes, and delivering molecular oxygen (O_2_) to cells (Ryter and Tyrrel, 2000). Heme is presented in different forms, mostly in forms *a*, *b*, and *c*. Heme *a* and *c* are important in the physiology of electron transport and are found in cytochrome *c* oxidase (Tsukihara et al., 1995) and cytochrome *c* reductase (Xia et al., 1997; Zhang et al., 1998), respectively. Heme *b* is the most common type in mammals and is part of several proteins (Larsen et al., 2012) including the gas carriers hemoglobin (Hb; Park et al., 2006) and myoglobin (Evans and Brayer, 1988; Vojtechovsky et al., 1999), hemeproteins related to heme release during hemolysis and tissue damage. Importantly, heme *b* interaction with heme oxygenase (HO; Lad et al., 2003), the enzyme responsible for heme intracellular catabolism, and hemopexin (Hx; Paoli et al., 1999), a plasmatic heme scavenger, is essential for the regulation of free heme availability and Fe recycling (Kovtunovych et al., 2010; Tolosano et al., 2010).

Besides its physiological importance, heme has a potent oxidative capacity oxidizing lipids (Tappel, 1953, 1955; Vincent et al., 1988) and proteins (Aft and Mueller, 1984; Vincent, 1989), and damaging DNA (Aft and Mueller, 1983). Thus, heme can be a dangerous molecule once released from hemeproteins. Some diseases are characterized by high amounts of hemeproteins out from their physiological environments. The consequences of heme toxicity can be appreciated in hemolytic diseases such as β-thalassemia, sickle-cell disease (SCD), ischemia-reperfusion (IR), and malaria (Katori et al., 2002; Pamplona et al., 2007; Vinchi et al., 2013). Extracellular and intracellular proteins have essential functions controlling free heme availability, guarding tissues from its deleterious effects. Through evolution mammals acquired several protective mechanisms against heme toxicity. Under physiologic conditions or mild to moderate hemolysis, haptoglobin (Hp) binds Hb (Melamed-Frank et al., 2001). Because of its large molecular size, this complex is maintained in the intravascular space, preventing the association of otherwise free Hb with nitric oxide (NO; Reiter et al., 2002) and inhibiting the release of free heme (Melamed-Frank et al., 2001). The complex Hp:Hb binds to CD163 (Kristiansen et al., 2001), present in macrophages and hepatocytes (Philippidis et al., 2004; Quaye, 2008), which mediates the endocytosis of Hb–Hp complexes for degradation. However, during hemolytic diseases high amounts of Hb are released in the intravascular environment, saturating the Hp molecules and thus accumulating free Hb (Muller-Eberhard, 1970). In the presence of reactive oxygen species (ROS), Hb is oxidized to methemoglobin (MetHb; Balla et al., 1993), characterized by the change in the oxidative state of the Fe present in the heme molecule from ferrous (Fe^+2^) to ferric (Fe^+^^3^). MetHb is unstable and rapidly releases free heme (Balla et al., 1993). In this context, Hx scavenges free heme, binding it with very high affinity (Paoli et al., 1999). Hx inhibits the oxidative property of heme (Eskew et al., 1999) and mediates heme transportation to intracellular compartments through the macrophage receptor CD91 (Hvidberg et al., 2005), a critical step on heme catabolism. Once inside the cells, heme is catabolized by HO enzymes, generating equimolar amounts of biliverdin, carbon monoxide (CO), and Fe (Tenhunen et al., 1968). Differently from biliverdin and CO, which have anti-inflammatory effects (Otterbein et al., 2000; Baranano et al., 2002), free Fe is highly oxidative and can promote free radicals generation through the Fenton reaction, which catalyzes hydroxyl radicals from the reaction of Fe with H_2_O_2_ (Fenton, 1894). These radicals initiate lipid-chain peroxidation by obtaining electrons from other molecules, including unsaturated fatty acid (RH), generating an alkyl radical (R•; Ryter and Tyrrel, 2000). Thus, labile Fe can induce cytotoxic effects (Gozzelino et al., 2012). In this context, the HO function is complemented by ferritin, a ubiquitous intracellular protein complex that stores Fe in a soluble and non-toxic form (Eisenstein et al., 1991). Ferritin is composed of a heavy and a light chain which cooperates in the overall uptake and store of Fe in a non-toxic form protecting cells from hydroxyl radicals generated through the Fenton reaction (Arosio et al., 2009). While the ferritin heavy chain (FtH) presents a ferroxidase activity that converts the Fe from Fe^+^^2^ into Fe^+^^3^ (Broxmeyer et al., 1991), the ferritin light chain (FtL) is essential to promote Fe nucleation (Levi et al., 1992). Cells deficient on FtH are more susceptible to oxidative damage, while increased amounts of FtH protects cells from death induced by challenges such as Fe, tumor necrosis factor (TNF), heme, heme plus TNF, or oxidized low-density lipoprotein (LDL; Juckett et al., 1995; Pham et al., 2004; Gozzelino et al., 2012). All these effects require the ferroxidase activity of FtH.

Not only vertebrates need to manage high amounts of free heme. Hematophagous arthropods, upon digestion of Hb in the mid-gut, are exposed to enormous quantities of free heme and ROS. Different blood-sucking species have developed independent adaptations to deal with heme toxicity, including heme aggregation (Oliveira et al., 1999), heme degradation (Paiva-Silva et al., 2006; Pereira et al., 2007), expression of heme-binding proteins (Oliveira et al., 1995), and induction of antioxidant systems (Paes et al., 2001; Oliveira et al., 2011).

## DIRECT CYTOTOXIC EFFECTS OF HEME

The cytotoxicity of heme was initially associated with a direct effect due to its structural properties, specifically the capacity of the hydrophobic porphyrin ring to intercalate lipid layers and the presence of the atom of Fe, considered essential for heme-induced ROS generation. Heme can destabilize biological membranes increasing its permeability and the chance of lysis (Schmitt et al., 1993), as it happens with erythrocytes (Chiu and Lubin, 1989). This effect relies in the amphipathic properties of the porphyrin ring rendering heme the tendency to allocate in non-polar niches including lipoproteins and lipid membranes where it might establish dangerous contacts with lipids, important targets of peroxidation by ROS (Rose et al., 1985; Light and Olson, 1990a, b). Once intercalated into cellular plasma membranes heme amplifies cellular susceptibility to oxidative-mediated injury by oxidants such as H_2_O_2_ or those derived from activated inflammatory cells (Balla et al., 1991a, b, 1993). Because heme has an atom of Fe in its structure and can sensitize cellular membranes to H_2_O_2_, it was proposed that heme-induced cytotoxicity could be derived from free radicals generated through the Fenton reaction (Vincent, 1989; Chiu et al., 1996). However, conflicting data makes uncertain that heme can directly catalyze this reaction. Studies using electron paramagnetic resonance (EPR) spin-trapping techniques, in cell free conditions, suggested that neither MetHb (Mao et al., 1994), nor free heme (Van der Zee et al., 1996), can catalyze the Fenton reaction generating hydroxyl radicals. Furthermore, heme induces ROS generation dependent on enzymatic reactions. In fact, it was demonstrated that heme triggers ROS production by the NADPH oxidase enzyme (Graça-Souza et al., 2002; Arruda et al., 2004; Moraes et al., 2012; Barcellos-de-Souza et al., 2013) and by mitochondria (unpublished), through activation of specific signaling pathways (Graça-Souza et al., 2002; Porto et al., 2007; Fernandez et al., 2010; Fortes et al., 2012). Alternatively, heme-induced formation of radical species relies on the conversion of low-reactive organic hydroperoxides (ROOH) into highly reactive alkoxyl (RO•) and peroxyl (ROO•) radicals (Tappel, 1953, 1955; Van der Zee et al., 1996). These radicals may initiate further lipid peroxidation forming alkyl radicals that in the presence of O_2_ form more peroxyl radicals leading to a facile propagation of free radical reactions. Ultimately, alkyl radicals are rearranged to form toxic conjugated diens and stimulate the autocatalytic lipid peroxidation cascades (Ryter and Tyrrel, 2000) which could mediate heme-induced toxicity. Together, these data suggest that the cytotoxic heme effects can be mediated by ROS from different sources generated both through non-enzymatic and enzymatic reactions. This leads to at least four possible mechanisms of heme-induced ROS: (1) heme might directly promote ROS generation by the Fe present in its structure through the Fenton reaction, (2) Fe might be released from heme to exert its oxidative effects, (3) heme can activate signaling pathways to induce ROS generation enzymatically, or (4) heme can convert organic hydroperoxides into free radicals.

## INFLAMMATORY EFFECTS OF HEME

Heme activates innate immune cells and non-hematopoietic cells promoting inflammation. Heme injection in mice leads to vascular permeability, leukocyte migration from the intravascular environment to tissues and increase of acute-phase proteins (Lyoumi et al., 1999; Wagener et al., 2001b), hallmarks of acute inflammation. Heme activates endothelial cells inducing the expression of the adhesion molecules ICAM-1 (intercellular adhesion molecule 1), VCAM-1 (vascular cell adhesion molecule 1), E-selectin, P-selectin, and von Willebrand factor (VWF; Wagener et al., 1997; Belcher et al., 2014) and causes neutrophil migration (Graça-Souza et al., 2002; Porto et al., 2007). The adhesion molecules enable neutrophils to attach firmly to the endothelium and migrate to tissues parenchyma. Thus, leukocytes must be activated and express adhesion molecules complementary to those expressed by the endothelium. A seminal study demonstrated that the ability of heme to activate neutrophils depend on protein kinase C (PKC) activation and ROS generation, inducing the expression of adhesion molecules and modifying actin cytoskeleton dynamics, necessary features for neutrophils migration (Graça-Souza et al., 2002). In fact, heme can induce neutrophil migration by acting as a chemotactic molecule (Porto et al., 2007) or by inducing the production of leukotriene B4 (LTB4) by macrophages (Monteiro et al., 2011). Heme-induced neutrophils activation leads to extracellular traps (NETs) release through a mechanism dependent on ROS generation (Chen et al., 2014). Moreover, heme induces the production of the chemokine interleukin (IL)-8 by endothelial cells (Natarajan et al., 2007) and by neutrophils (Graça-Souza et al., 2002). Although neutrophils have important functions controlling infection, these cells can promote vascular and tissue injury by generating ROS, secreting proteases, and releasing extracellular chromatin (NETs; reviewed in Mócsai, 2013). Consequently, the prolonged activity of these cells is detrimental for tissue homeostasis. In this context, heme inhibits neutrophils apoptosis, increasing their longevity, and possibly enhancing harmful stimuli from these cells (Arruda et al., 2004, 2006).

Macrophages stimulate inflammatory responses by secreting cytokines, chemokines, and lipid mediators. TNF, IL-6, and IL-1β are pleiotropic cytokines that regulate cell death, increase vascular endothelial permeability, recruit immune cells to inflamed tissues and induce the production of acute-phase proteins. Part of these effects is mediated by the activation of fibroblasts and endothelial cells, especially by TNF and IL-1β. Moreover, IL-1β and TNF modifies the hypothalamus threshold of the body temperature causing fever. KC (keratinocyte-derived chemokine) is a chemokine that attracts neutrophils to sites of inflammation and LTB4 is a lipid mediator that functions as a chemoattractant molecule and also activates leukocytes. Heme activates macrophages inducing the production of TNF, KC (Figueiredo et al., 2007), IL-1β (unpublished), and LTB4 (Monteiro et al., 2011). Although KC and IL-1β functions were not investigated during heme-induced inflammatory effects, TNF and LTB4 were described as essential inflammatory mediators during inflammatory events induced by heme. As described before, LTB4 has an important function regulating heme-induced neutrophils migration (Monteiro et al., 2011). On the other hand, TNF secretion induced by heme is essential for the activation of the programed necrotic cell death pathway, which is denominated necroptosis (Fortes et al., 2012).

Hemoglobin is used as a nutrient by *Plasmodium*, the protozoan parasite that causes malaria, during its replication stage inside erythrocytes in the course of the infection in mammals (Francis et al., 1997). Hb digestion generates high amounts of free heme which is detoxified by its polymerization into a crystal structure named hemozoin (Hz; Slater et al., 1991; Slater, 1992; Pandey and Tekwani, 1996). Once released from ruptured erythrocytes, Hz can be phagocytized and accumulated inside macrophages altering their functions. Similarly to heme, Hz also induces the production of several inflammatory mediators by macrophages such as cytokines and chemokines, and induces leukocytes migration (reviewed by Olivier et al., 2014). In nature, Hz produced by *Plasmodium* spp. carries DNA from the parasite. Because DNA is immunogenic and can activate innate immune receptors (Yanai et al., 2009; Coban et al., 2010), the exact role of pure Hz in the activation of innate immunity is not completely understood (discussed later).

Together, these inflammatory responses triggered by heme indicate that heme is a damage-associated molecular pattern (DAMP), a group of endogenous molecules derived from damaged cells and extracellular matrix degradation capable of promoting and exacerbating immune responses. These concepts challenged the idea that the cytotoxic and inflammatory effects of heme were exclusively mediated by the oxidative capability of the Fe associated with the amphipathic property of the porphyrin ring. Moreover, albeit the controversy about Hz immunogenicity, Hz composed of heme and *Plasmodium* DNA can be defined as a pathogen-associated molecular pattern (PAMP) in the course of malaria infection, promoting inflammation and contributing to pathogenesis.

## HO-1 PROTECTION AGAINST HEME

Plasma scavengers of heme and/or Hb once saturated allow heme to exert its inflammatory properties. In fact, hemolysis or heme injection in *Hx*^-/-^ mice cause increased inflammation and severe renal damage compared to wild type (WT) mice (Tolosano et al., 1999; Vinchi et al., 2008). Mice lacking HO-1 (*Hmox1*^-/-^) are highly susceptible to pathologic conditions associated with increased serum heme concentration. For instance, *Hmox*^-/-^ mice develops acute renal failure and marked mortality when submitted to rhabdomyolysis, a pathological condition that increases serum myoglobin which can be oxidized and release heme (Nath et al., 2000). Furthermore, *Hmox1*^-/-^ mice are susceptible to liver IR which is characterized by tissue damage in sites that are reperfused after ischemia injury and hemolysis (Devey et al., 2009). Thus, HO-1 regulation by heme is an essential feedback mechanism that maintains tissue homeostasis through anti-inflammatory and anti-oxidant activities in pathologic situations associated to heme (reviewed by Gozzelino et al., 2010). SCD and β-thalassemia are genetic diseases associated to erythrocytes that are prone to lysis due to defective Hb production (Heinle and Read, 1948; Pauling et al., 1949; Ingram, 1957; discussed later). Asymptomatic humans with sickle-cell trait are protected against malaria disease. Interestingly, this fact was suggested to be dependent on heme-induced HO-1 expression. Transgenic sickle-cell hemizygous mice are protected against malaria disease (Ferreira et al., 2011). Besides the fact that these mice do not develop anemia, hemolysis is evidenced by a modest serum heme increase which is sufficient to increase HO-1 expression and CO production (Ferreira et al., 2011). CO inhibits Hb oxidation and subsequently heme release, thus blocking heme accumulation in serum and preventing heme from exerting its inflammatory effects in the course of malaria disease (Ferreira et al., 2011). This case demonstrates the importance of the feedback production of HO-1 induced by heme to maintain tissue homeostasis. Moreover, heme accumulates in wounded areas and promotes inflammation through the expression of adhesion molecules and the recruitment of leukocytes (Wagener et al., 2003a). During the resolution phase of inflammation HO-1 expression in leukocytes reduces adhesion molecules expression and leukocytes migration, thus contributing to wound healing (Wagener et al., 2003a). Although heme is a pro-inflammatory molecule, the feedback activity of HO-1 inhibits exacerbated inflammation and maintains homeostasis. However, the efficiency of HO-1 to counteract heme depends on the health state of the individual. Heme administration in mice without a genetic or pathologic condition that cause susceptibility to heme might have a therapeutic effect dependent on the activity of HO-1. In fact, heme-induced HO-1 expression prevents injury induced by indomethacin in the intestine (Yoriki et al., 2013), unilateral ureteral obstruction in the kidney (Correa-Costa et al., 2010), nutricional steatohepatitis (Nan et al., 2010), and IR in the liver (Xue et al., 2007).

## INNATE IMMUNE RECEPTORS ACTIVATED BY HEME

The immune system is characterized by the exquisite recognition of exogenous and endogenous molecules, and by its central role in tissue homeostasis as well as contributing to the pathogenesis of several diseases. The physiological consequence of an infection is the activation of an immune response essential to pathogen control or elimination (Medzhitov, 2008). Similarly, sterile tissue damage triggers inflammatory responses fundamental to tissue repair (Nathan and Ding, 2010). For years the mechanism of innate cell activation and function was considered non-specific, in opposition to the ability of B and T lymphocytes to recognize antigens by clonal receptors. The specific recognition of molecules from infectious agents and from endogenous origin by innate immune cells is performed by evolutionary conserved membrane and cytosolic receptors also known as pattern recognition receptors (PRRs; reviewed in Takeuchi and Akira, 2010). The presence and function of these receptors on mammals were originally proposed by Janeway (1989) and first confirmed by his group (Medzhitov et al., 1997). PRRs are germline-encoded and represent an essential arm of innate immune cells to sense invading microbes through conserved PAMPs and to detect endogenous stress signals through DAMPs. Several families of innate immune receptors have been described in recent years.

### HEME TRANSPORTERS

For decades it has been appreciated that the transport of nutritional heme across cellular membranes of intestinal epithelial cells is an active and regulated process, more efficient than free Fe transport, requiring energy, and a selective transporter. The molecular nature of mammalian heme transporters remained elusive for many years, in part due to the lack of molecular and genetic tools and also as a consequence of a number of membrane proteins able to bind and transport heme at low affinity. In fact, several transporters of heme and other porphyrins have been described in recent years, although the *in vivo* importance of some of these molecules on heme trafficking is still object of debate. The cytoplasmic heme importer, named heme carrier protein 1 (HCP1), is expressed in the cytoplasmic membrane of duodenal epithelial cells and is believed to participate on dietary heme absorption (Shayeghi et al., 2005; Latunde-Dada et al., 2006). It was also demonstrated the critical *in vivo* importance of this putative heme transporter as a mediator of extracellular folate import by intestinal epithelial cells (Qiu et al., 2006). Similarly, the ATP-binding cassette, Abcg2, involved on heme export is also associated with multidrug resistance (Krishnamurthy et al., 2004; Krishnamurthy and Schuetz, 2006). A second exporter of heme is the feline leukemia virus subgroup C receptor 1 (FLVBR1), in this case the lack of the transporter in mice affects erythroid differentiation and cell survival (Quigley et al., 2004; Keel et al., 2008). A mitochondrial isoform of FLVCR1 named FLVCR1b was recently demonstrated to export heme from the mitochondria to the cytosol (Chiabrando et al., 2012). The heme transporter ATP-binding cassette, Abcb6, controls the translocation of protoporphyrin between the cytosol and the mitochondria (Krishnamurthy et al., 2006). The heme-responsive gene-1 (HRG1) participates in the translocation of heme between the cytosol and endosomal compartments (Rajagopal et al., 2008).

An interesting study demonstrated that heme binds with high affinity to a heme-binding motif present at the intracellular domain of Slo1 channels, and inhibits transmembrane K currents by decreasing the frequency of channel opening (Tang et al., 2003). This was the first demonstration of an acute signaling effect of heme dependent on a specific transmembrane protein. Heme interactions with these membrane proteins led us to hypothesize that heme activates cells of the innate immune system through binding to an innate immune receptor.

### HEME IS AN AGONIST OF TOLL-LIKE RECEPTOR 4

Using mouse strains deficient in different adaptors and innate immune receptors, we observed that heme-induced TNF production was dependent on MyD88 and Toll-like receptor 4 (TLR4; Figueiredo et al., 2007). TLRs are receptors present in mice and humans, which are distributed among the cellular membrane and intracellular compartments, as endosomes and lysosomes (Takeuchi and Akira, 2010). Each TLR recognize one or more PAMPs derived from different microbes such as bacteria, fungi, parasites, and virus (Takeuchi and Akira, 2010). Besides microbial agonists, TLRs triggers inflammatory responses to endogenous inflammatory molecules. DAMPs that activate TLRs include the high-mobility group box 1 (HMGB1) nuclear protein, myeloid-related proteins (Mrp8/14), amyloid β, oxidized LDL (oxLDL), DNA, and RNA in the form of immune complexes, mitochondrial DNA (Oka et al., 2012) and extracellular matrix components such as tenascin-C, fibronectin, and hyaluronan (reviewed by Yu et al., 2010). Other DAMPs such as ATP, formyl peptides, uric acid crystals, and cholesterol crystals activate other innate immune receptors (Mariathasan et al., 2006; Martinon et al., 2006; Duewell et al., 2010; discussed later).

**FIGURE 1:**
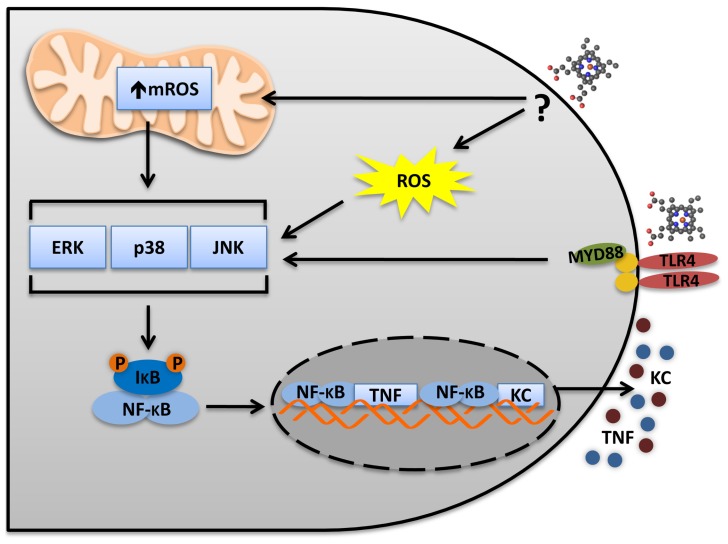
**Heme activates TLR4 in macrophages.** The TLR4 activates two distinct pathways: MyD88 and TRIF. In macrophages, heme induces a biased MyD88 activation and the secretion of the pro-inflammatory cytokines TNF and KC. TLR4 activation leads to MAPKs and NFκB activation, which are necessary to TNF secretion. Heme induces ROS generation independently of TLR4. However, ROS is necessary to induce TNF secretion and MAPK activation. Thus, TLR4 activation and ROS generation seem to be complementary and both are required for MAPKs and IκB degradation. It will be important to determinate the source of ROS and the mechanisms triggering heme-induced ROS generation. The mitochondria and the NADPH oxidase complexes seem to be involved. mROS scavenger (Mito-TEMPO) and NADPH oxidases inhibitors (apocynin and DPI) block TNF production induced by heme.

A critical step in characterizing an endogenous molecule as an agonist of an innate immune receptor is the necessity to exclude the presence of any microbial contaminant. In fact, in the case of heat shock proteins (HSPs), the effect on TNF secretion was later attributed to endotoxin contamination in the protein preparations (Gao and Tsan, 2003). Several strategies were used to exclude that the TLR4-induced activation by heme was due to contaminant (Figueiredo et al., 2007). Highly purified heme free of endotoxin contamination was used, as well as polymyxin B, anti-TLR4/MD2, and lipid A antagonist, all of which inhibited the effects of LPS but did not interfere with the induction of TNF by heme. Similarly, protoporphyrin IX (PPIX), an antagonist of heme, did not inhibit the activity of LPS on TNF secretion (Figueiredo et al., 2007). The TLR4 engagement by LPS triggers two distinct pathways by the recruitment of the adaptor molecules MyD88 (Muzio et al., 1997; Wesche et al., 1997) and TRIF [TIR (Toll–IL-1 receptor)-domain-containing adapter-inducing interferon (IFN)-β; Hoebe et al., 2003; Yamamoto et al., 2003]. The MyD88-dependent pathway leads to the activation of mitogen-activated protein kinases (MAPKs) and NF-κB (nuclear factor kappa-light-chain-enhancer of activated B cells) transcription factors inducing the expression of inflammatory cytokines such as TNF, IL-6, IL-1β, and KC (Takeuchi and Akira, 2010). The TRIF-dependent pathway activates the IRF3 (IFN regulatory transcription factor-3), which induce the expression of type I IFN, and late-phase NF-κB (Takeuchi and Akira, 2010). Heme seems to activate only the MyD88-dependent pathway and is ineffective to induce several cytokines or the expression of co-stimulatory molecules typical of TLR4 activation by LPS. It can induce MAPK activation and the expression of TNF and KC, but not of IFNβ or the IFN-dependent gene IP-10 (Figueiredo et al., 2007; **Figure 1**). Even the transcription of certain MyD88-dependent genes is not induced by heme when compared to LPS. In fact, different TLR4 ligands trigger signaling pathways differently. While, monophosphoryl lipid A, an analog of the TLR4 agonist tetra-acylated lipid A, induces a biased response to the TRIF pathway (Mata-Haro et al., 2007), the vesicular stomatitis virus glycoprotein G induces a biased response to the TRAM pathway to specifically induce IRF7 activation (Georgel et al., 2007). Moreover, human embryonic kidney (HEK) cells transfected with human TLR4 secretes IL-8 upon stimulation with heme (Piazza et al., 2011). In this case, the experiments were performed in the absence of CD-14 and MD-2 transfection.

The differences in signaling pathways activated by different agonists of the TLR4 might be due to (1) a different binding site on the TLR4, compared to LPS, or perhaps (2) the involvement of different co-receptors. (1) Molecular determinants of heme structure for TLR4 activation were determined (Figueiredo et al., 2007). Heme (Fe-PPIX) analogs, porphyrin rings without Fe (PPIX) or with metal substitutions (Pd-PPIX and Sn-PPIX), do not induce TNF production. Fe-mesoporphyrin IX, a heme analog with two ethyl groups substituting its vinyl groups, also is unable to activate TLR4. These data suggests that coordinated Fe and the vinyl groups are necessary for heme effect on TNF production. PPIX functions as an antagonist for TNF production induced by heme but not by LPS (Figueiredo et al., 2007). Conversely, LPS antagonists do not inhibit TNF production induced by heme. Interestingly, the sequence comparison of TLR4 and globins from different species suggests the presence of conserved heme-binding sequences in TLR4 (unpublished). These conserved sequences present a histidine which is essential for heme to bind globins. It is tempting to speculate that this histidine is essential for heme induced TLR4 activation. In fact, this same conserved histidine, together with two others non-conserved histidines, was shown to be important for the TLR4 activation induced by nickel in humans cells (Schmidt et al., 2010). The lack of these non-conserved histidines in the murine TLR4 disables the nickel capacity to activate this receptor. Moreover, this conserved histidine is important to Nickel, but not to LPS, to activate the TLR4. (2) Depending on the TLR4 activator, different co-receptors will be involved. LPS depends on MD2 and CD14 to activate TLR4 (Park et al., 2009). Amyloid β and oxLDL induce the assembly of a CD36–TLR4–TLR6 complex (Stewart et al., 2010), while hyaluronan is recognized by a complex formed by TLR4, MD2, and CD44 (Taylor et al., 2007). Heme depends on CD14 but not MD2 to activate TLR4 and induce TNF (Figueiredo et al., 2007; Piazza et al., 2011).

The crystal structure formed by heme polymerization, Hz, has pro-inflammatory properties dependent on TLR9 activation. However, it is uncertain whether pure Hz can directly activate TLR9. Because Hz isolated from *P. falciparum* cellular extract may contain DNA, this might be the TLR9 activator. In fact, it was shown that DNase treatment of *Plasmodium* Hz abolishes cytokine production by dendritic cells (DCs; Parroche et al., 2007). However, there is evidence that synthetic Hz, which is free of DNA, induces TLR9-dependent responses in DCs (Coban et al., 2005). Moreover, synthetic Hz induces conformational changes in recombinant TLR9, similar to those induced by CpG DNA, a well characterized TLR9 agonist (Coban et al., 2010). Interestingly, heme was able to induce similar conformational changes in the recombinant TLR9 (Coban et al., 2010). However, we demonstrated that TLR9 was not involved in heme-induced TNF production because WT and *Tlr9*^-/-^ macrophages produce similar amounts of TNF when stimulated with heme (Figueiredo et al., 2007). Moreover, this demonstration suggests that mitochondrial DNA, a TLR9 activator (Oka et al., 2012), is not involved in the production of TNF by heme.

### G PROTEIN-COUPLED RECEPTORS

As mentioned before, heme induces LTB4 production by macrophages to stimulate neutrophil migration *in vivo* (Monteiro et al., 2011). Surprisingly, heme can also induce neutrophil migration *in vitro* thus indicating that heme acts as a chemotactic molecule (Graça-Souza et al., 2002; Porto et al., 2007). Chemokines and chemotactic molecules can induce neutrophil migration through G protein-coupled receptors (GPCR) which are sensitive to pertussis toxin (reviewed in Rot and von Andrian, 2004). Pertussis toxin inhibits heme-induced ROS generation and neutrophil migration, thus suggesting the involvement of a GPCR (Porto et al., 2007). Pharmacological inhibition of several pathways activated by GPCR [PI3K, PKC, phospholipase C (PLC)β, Rho, p38] demonstrated that ROS generation and neutrophil migration depends on the activation of signaling pathways characteristic of chemotactic receptors (Porto et al., 2007). Interestingly, PI3K and MAPK activation delays neutrophil apoptosis through a NADPH oxidase-dependent mechanism (Graça-Souza et al., 2002). This indicates that similar pathways might be involved in ROS generation, survival, and migratory mechanisms triggered by heme in neutrophils. Furthermore, although several heme analogs were able to induce neutrophil migration, mesoporphyrins, molecules lacking the vinyl groups in their rings, were not chemotactic for neutrophils and selectively inhibited the migration induced by heme (Porto et al., 2007). We believe that mesoporphyrins functions as antagonist molecules blocking the activation of an extracellular receptor. Cytochrome *c* has a conserved sequence responsible for its interaction with heme. This same sequence is present in the Slo1 channel and was proved to be essential for its binding to heme (Tang et al., 2003). Interestingly, we found this conserved sequence in two chemotactic receptors expressed in neutrophils. Together, these results raises the possibility that heme activates neutrophils through the activation of a GPCR.

## MECHANISMS INVOLVED IN HEME-INDUCED CELL DEATH AND INFLAMMATION

### CYTOTOXIC EFFECTS CONTROLLED BY SPECIFIC SIGNALING PATHWAYS

**FIGURE 2:**
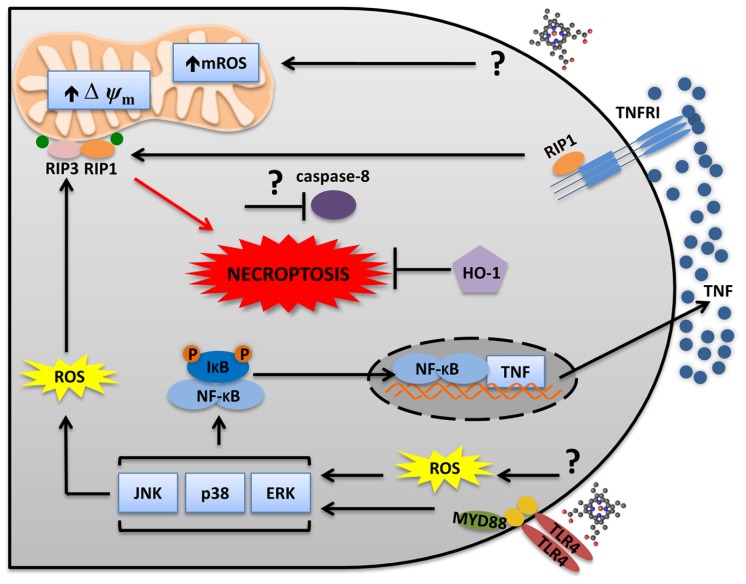
**Heme induces RIP1/RIP3-dependent programed cell death.** Heme triggers two distinct but integrated pathways to promote necroptosis. Heme induces ROS generation independently of TLR4. ROS sensitizes macrophages to TNFR-induced cell death. Heme-induced TLR4 activation leads to MAPKs activation and TNF production. While JNK increases ROS generation, TNF induces RIP1–RIP3 necrosome which triggers necroptosis. The mitochondria and the NADPH oxidase complexes seem to be involved. mROS scavenger (Mito-TEMPO) and NADPH oxidases inhibitors (apocynin and DPI) block heme-induced necroptosis. Necroptosis only develops when caspases are inhibited. However, it is not known if heme requires caspase inhibition to induce necroptosis. Moreover, HO-1 has a protective effect during heme-induced necroptosis.

Because of its lipid intercalating ability, heme was assumed to induce its cytotoxic and inflammatory effects mediated by its pro-oxidant and amphipathic properties. However, new possibilities emerged because of the new characterized mechanisms of inflammatory responses induced by heme. Heme activates TLR4 to induce cytokine production and necrotic cell death (Figueiredo et al., 2007; Fortes et al., 2012; **Figure 2**). Heme-induced necrosis depends on TNF produced by TLR4 activation, because *Tlr4*^-/-^ and *Tnfr*^-/-^ macrophages are resistant to the cytotoxic effects of heme (Fortes et al., 2012). However, heme sensitizes *Tlr4*^-/-^ macrophages to TNF-induced cell death, indicating that TLR4 is essential to heme-induced TNF production, but not directly required for the cell death induced by heme. The necrotic cell death induced by heme was dependent on ROS generation, since antioxidants prevented it (Fortes et al., 2012). Moreover, *Rip1*^-/-^ and *Rip3*^-/-^ cells were resistant to heme-induced necrotic cell death (Fortes et al., 2012), characterizing the type of programed cell death as necroptosis (Cho et al., 2009; He et al., 2009; Zhang et al., 2009). Interestingly, ROS induction is essential but not sufficient to cause cell death. In fact, TLR4 and TNFR deletions do not change the capacity of heme to induce ROS generation (Figueiredo et al., 2007). Therefore, it is clear that heme triggers ROS-induced sensitization of macrophages to TNFR-mediated cell death through RIP1 and RIP3 activation to promote necroptosis (**Figure 2**). Moreover, inhibition of Jun N-terminal kinase (JNK) also decreases heme-induced ROS and necroptosis (Fortes et al., 2012; **Figure 2**). Heme-induced necroptosis is reversed by deferoxamine, a Fe chelator. Fe can mediate non-enzymatic ROS generation by the Fenton reaction. However, since deferoxamine binds to heme (Baysal et al., 1990; Lu et al., 2012), we cannot exclude the possibility that its inhibitory effect is directly related to heme and not to free Fe. In fact, the protective effect of pharmacologic inhibition of NADPH oxidase and mitochondrial ROS (mROS) on heme-induced TNF and macrophage necroptosis suggests that enzymatic ROS production is essential for the cytotoxic effects of heme (unpublished).

Heme also induces programed cell death in non-hematopoietic cells. Stimulation of astrocytes with heme, as a model of hemorrhagic injury, causes cell death with characteristics of programed necrosis including the loss of plasma membrane integrity, reversion by necrostatin-1, a selective inhibitor of RIP1, and by antioxidants (Laird et al., 2008). Extracellular-signal-regulated kinase (ERK) activation is also involved in astrocyte cell death induced by heme (Regan et al., 2001). Interestingly, heme-induced ERK activation in these cells is ROS-independent (Regan et al., 2001) indicating the involvement of a distinct pathway that might involve TLR4 signaling. In fact, TLR4 is involved in intracerebral hemorrhage (ICH) induced by heme (Lin et al., 2012). Microglia cells, another cell type present in the central nervous system, undergoes cell death through heme-induced activation of JNK and p38 (Cai et al., 2011). Moreover, heme induces apoptosis in human brain vascular endothelial cells (HBVEC) by STAT3 (signal transducer and activator of transcription 3)-dependent activation of matrix metallopeptidase 3 (MMP3; Liu et al., 2013). Heme also sensitizes other non-hematopoietic cells to TNF-induced cell death (Seixas et al., 2009; Larsen et al., 2010; Sukumari-Ramesh et al., 2010; Gozzelino et al., 2012). This cell death has morphological and biochemical characteristics of apoptosis including caspase activation, membrane blebbing, nuclear shrinking, and fragmentation, as well as chromatin condensation and formation of apoptotic bodies (Seixas et al., 2009). Treatment with antioxidants, increased expression of HO-1 or Hx also revert the heme/TNF-induced cell death in hepatocytes (Seixas et al., 2009; Gozzelino et al., 2010, 2012; Larsen et al., 2010; Gozzelino and Soares, 2011).

Heme induces HO-1 that in turn protects cells from death (Vile et al., 1994; Poss and Tonegawa, 1997a; Ferris et al., 1999; Brouard et al., 2002; Arruda et al., 2004; Seixas et al., 2009; Gozzelino et al., 2010; Kovtunovych et al., 2010; Larsen et al., 2010). The ability of HO-1 to promote cytoprotection has been attributed to its role controlling heme amounts as well as Fe homeostasis and oxidative stress (Gozzelino et al., 2010). Mice lacking HO-1 (*Hmox1*^-/-^) has Fe overload, increase cell death and widespread inflammation (Poss and Tonegawa, 1997a, b; Kovtunovych et al., 2010). *In vitro* embryonic fibroblasts from *Hmox1*^-/-^ mice are more sensitive to heme or H_2_O_2_ and present increase oxidative stress (Poss and Tonegawa, 1997a). Moreover, *Hmox1*^-/-^ mice have reduced macrophage numbers in the spleen and liver due to cell death (Kovtunovych et al., 2010). This increased macrophage cell death was confirmed *in vitro* upon erythrophagocytosis, further suggesting that the *in vivo* exposure to heme is the cause of the increased macrophage death observed in *Hmox1*^-/-^ mice. This result is corroborated by the increased necroptosis observed in *Hmox1*^-/-^ macrophages exposed to heme compared to WT (Fortes et al., 2012). Treatment with antioxidants fully blocked heme-induced cell death of *Hmox1*^-/-^ macrophages.

Importantly, increased expression of FtH also protects different cell types from the cytotoxic effects of heme, TNF or heme and TNF (Balla et al., 1992; Berberat et al., 2003; Cozzi et al., 2003; Gozzelino et al., 2012). These results suggest that Fe derived from heme catabolism participates in the heme-induced sensitization to cell death. The mechanism depends on the sustained activation of JNK induced by Fe-derived ROS. The increased JNK activation inhibits FtH expression, increasing the amount of labile Fe and consequently the amounts of ROS. The antioxidant property of FtH blocks TNF-induced JNK activation, reducing cell death (Pham et al., 2004; Kamata et al., 2005; Gozzelino et al., 2012). Thus, it seems that JNK activation is a common trigger of heme-induced cell death in a variety of cells (macrophages, hepatocytes, and microglia; **Figure 2**). Thus heme induces programed cell death in different types of cells by the regulation of specific signaling pathways.

### THE SPLEEN TYROSINE KINASE PATHWAY OF INFLAMMATION

The cytotoxic effect of heme has important impact in different pathological conditions. This is in part due to the loss of essential tissue functions and in part due to the release of cellular contents with inflammatory activities. High amounts of free heme are found in infectious diseases, such as malaria and sepsis (Pamplona et al., 2007; Larsen et al., 2010), suggesting that heme can amplify inflammatory responses during infection. In fact, it was demonstrated that dying hepatocytes release HMGB1 upon heme and TNF challenge, which in turn increases the systemic inflammatory response (Larsen et al., 2010). Another mechanism involved in heme-induced inflammation during infectious conditions is the amplification of cytokine production induced by heme with microbial molecules. Hb synergizes with LPS enhancing the production of pro-inflammatory cytokines by macrophages (Yang et al., 2002). The opposite effect of globin indicates that heme moiety is the responsible for the potentiating effect of Hb. A low dose of LPS on its own that causes an insignificant cytokine secretion, together with heme results in substantial production of cytokines, thus suggesting that this synergism may be particularly important under conditions of low agonist concentrations, helping the control of infectious agents (Fernandez et al., 2010). This amplification response can also be deleterious under higher concentrations of heme and microbial molecules. *In vivo*, injection of heme and LPS induces a significant increase in the concentrations of TNF and IL-6 when compared to the challenge with LPS alone (Fernandez et al., 2010). Moreover, the co-injection of non-lethal doses of heme and LPS induces 100% lethality.

**FIGURE 3:**
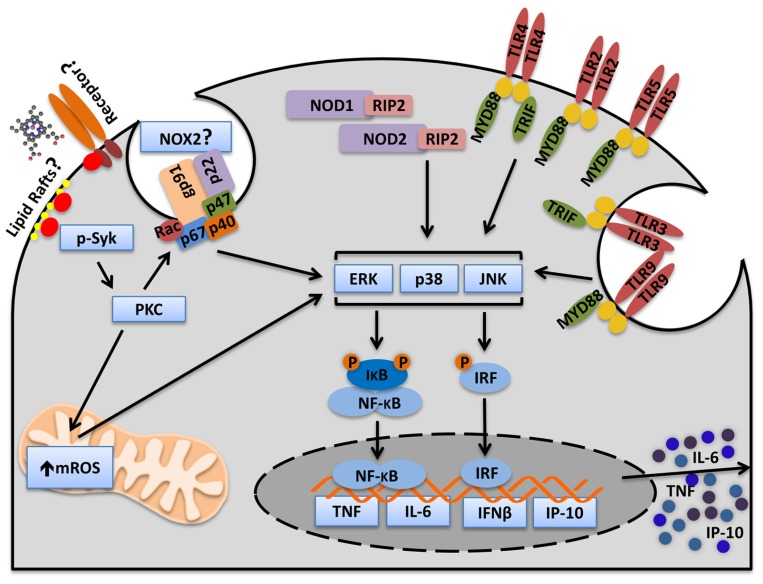
**Heme activates Syk and amplifies cytokine production induced by PAMPs.** Heme induces Syk phosphorylation in macrophages. The Syk pathway is essential for heme-induced ROS production. Heme-induced ROS generation increases MAPKs and NFκB activation and consequently, cytokine production. Heme amplifies cytokines induced by cell surface receptors (TLR2, TLR4, TLR5), endosome receptors (TLR3, TLR9), and cytosolic receptors (NOD1 and NOD2). Moreover, heme amplifies MyD88- (TNF and IL-6) and TRIF-dependent (IP-10) cytokines. The mechanism by which heme triggers Syk activation is not known. However, there are two possibilities. Heme could trigger Syk activation through an unknown surface receptor or through interaction with lipid rafts. The source of ROS controlled by Syk is not known but because PKC, NOX, and mitochondria inhibitors blocks the synergism between heme and PAMPs it is possible to consider that Syk controls ROS generation by NOX and mitochondria. Although NOX2 is the principal NADPH oxidase complex in phagocytes we cannot exclude the possibility that other NOX might be involved.

Interestingly, although purified Hx inhibits the synergy between heme and PAMPs, the synergy only occurs in the presence of serum, a condition that protects the cells against heme-induced TLR4-dependent TNF production and necroptosis. Heme synergizes with ligands of TLR2, TLR3, TLR4, TLR9, NOD1 (nucleotide-binding oligomerization domain 1), and NOD2 to increase TNF and IL-6 production by macrophages (**Figure 3**). The synergy between heme and LPS, a TLR4 activator, is induced by the enhanced activation of MAPKs (ERK, p38, JNK) and NF-κB (Fernandez et al., 2010; **Figure 3**). The mechanism depends on spleen tyrosine kinase (Syk) phosphorylation which regulates the ROS production induced by heme (**Figure 3**). Heme-induced Syk phosphorylation modulates ROS generation by activating PKC and calcium signaling through PLCγ, two downstream molecules regulated by Syk, which are involved in the synergy between heme and LPS (Fernandez et al., 2010). In fact, Syk modulates signaling pathways activated by TLRs cytokine production by macrophages and DC (Brown et al., 2003; Gantner et al., 2003; Turnbull et al., 2005). Syk is a signaling molecule activated by receptors that signal through immunoreceptor tyrosine-based activation motifs (ITAMs) suggesting the involvement of a receptor in the synergy between heme and PAMPs (Underhill and Goodridge, 2007; **Figure 3**). On the other hand, Syk is also activated independently of cell surface receptors by disturbances in lipid rafts domains (Ng et al., 2008; **Figure 3**). Because of heme’s capacity to intercalate cellular membranes we cannot exclude the possibility that Syk might be activated by the direct contact of heme with cellular membranes. It will be important to investigate whether heme-induced Syk phosphorylation requires a receptor.

### THE NLRP3 INFLAMMASOME

While TNF and IL-6 are mainly regulated at the transcriptional and translational levels, cytokines such as IL-1β and IL-18 requires two steps to be produced. These cytokines are expressed as zymogens, pro-IL-1β and pro-IL-18, which are regulated by the synthesis of their mRNA by TLRs. However, IL-1β and IL-18 maturation requires cleavage of their zymogens by caspase-1 (Cerretti et al., 1992; Thornberry et al., 1992), which is activated independently of TLR signaling. The cleavage of these pro-forms is necessary for their biological effects. Caspase-1 is activated by a multienzymatic platform called the inflammasome, which is composed of NLRs (NOD-like receptors), ASC (apoptosis-associated speck-like protein), and caspase-1 (Martinon et al., 2002; Wen et al., 2013). NLRP3 (nucleotide-binding domain, leucine rich family, pyrin containing 3 gene) is the best characterized inflammasome receptor and is activated by several DAMPs such as ATP, Hz, amyloid β, uric acid crystals, and cholesterol crystals (Mariathasan et al., 2006; Martinon et al., 2006; Halle et al., 2008; Shio et al., 2009; Duewell et al., 2010; mechanisms reviewed in Latz et al., 2013). Three mechanisms are involved in the NLRP3 inflammasome activation: K^+^ efflux (Petrilli et al., 2007; Muñoz-Planillo et al., 2013), lysosome damage (Hornung et al., 2008), and mitochondrial dysfunctions that lead to mROS generation (Zhou et al., 2011) and the release of cardiolipin (Iyer et al., 2013) and mitochondrial DNA (Shimada et al., 2012).

In this context, we found that heme induces IL-1β production by activated LPS-primed macrophages promoting NLRP3-dependent processing of IL-1β (unpublished). Although heme is capable of activating TLR4, it does not induce IL-1β expression (Figueiredo et al., 2007). Heme (Fe-PPIX), but not its analogs, porphyrin rings without Fe (PPIX) or with metal substitutions (Pd-PPIX and Sn-PPIX), induces IL-1β processing and release (unpublished). The activation of NLRP3 by heme required K^+^ efflux, Syk phosphorylation, mROS, and NOX2. On the other hand, the mechanism was independent of ATP release and lysosomal damage (unpublished). Our results are in sharp contrast with a recent published report that describes the ability of heme to activate the NLRP3 inflammasome (Li et al., 2014). (1) Heme and PPIX caused the maturation of IL-1β in the absence of serum (Li et al., 2014). In our experimental conditions, heme did not activate the inflammasome in the absence of serum (unpublished). This same result was previously published (Dostert et al., 2009). Interestingly, the synergism of heme with PAMPs regarding the production of TNF and IL-6 is also dependent on serum (Fernandez et al., 2010; Lin et al., 2010). The reason why heme requires serum to induce part of its inflammatory effects needs further investigation. (2) Heme and PPIX primed the macrophages and induced IL-1β mRNA expression (Li et al., 2014). It is possible that the procedure to elicit peritoneal macrophages used in their study primed the cells for subsequent inflammasome activation. We observed that PPIX did not induce pro-IL-1β or inflammasome activation, suggesting that the iron in the porphyrin ring is essential for the effect of heme (unpublished). (3) Heme-induced IL-1β secretion depended on P2X receptors (Li et al., 2014). This observation suggests that ATP release is involved. In fact, ATP release from necrotic cells activates NLRP3 (Iyer et al., 2009). Importantly, their experimental procedure was made without serum. This could have increased the cell death of macrophages stimulated with heme and the subsequent released of ATP. Differently from their results, we demonstrated that heme induced similar amounts of IL-1β in WT and P2X7-deficient macrophages (unpublished). Moreover, the use of oxidized ATP (ATP antagonist) and apyrase (degrades ATP) corroborate that heme-induced inflammasome activation is independent of ATP (unpublished).

One of the mechanisms responsible for the NLRP3 activation involves the phagocytosis of crystal structures, subsequent lysosome damage and cathepsin B release to the cytosol (Hornung et al., 2008). In fact, phagocytosis and cathepsin B inhibition blocks the inflammasome activation induced by crystals, including Hz (Shio et al., 2009). Like heme, Hz also depends on ROS generation and Syk phosphorylation to activate NLRP3 (Shio et al., 2009). Heme, on the other hand, does not require phagocytosis or lysosome damage to induce IL-1β secretion (unpublished). Thus, heme activates the NLRP3 inflammasome with a distinct mechanism compared to ATP and Hz.

*In vivo*, heme injection induced IL-1β production and caspase-1-dependent neutrophil migration in mice peritoneum (unpublished). Moreover, *Caspase-1*^-/-^ mice were resistant to mortality induced by hemolysis. On the other hand, heme can be used to prevent inflammation through the induction of HO-1. In fact, preventive induction of HO-1 induced by heme inhibits the NLRP3 inflammasome activation in the liver (Kim and Lee, 2013) and the lung (Luo et al., 2014) in different pathological conditions.

## POSSIBLE ROLES OF HEME-INDUCED INNATE IMMUNE RECEPTOR ACTIVATION ON PATHOGENESIS

### SICKLE CELL DISEASE AND β-THALASSEMIA

**FIGURE 4:**
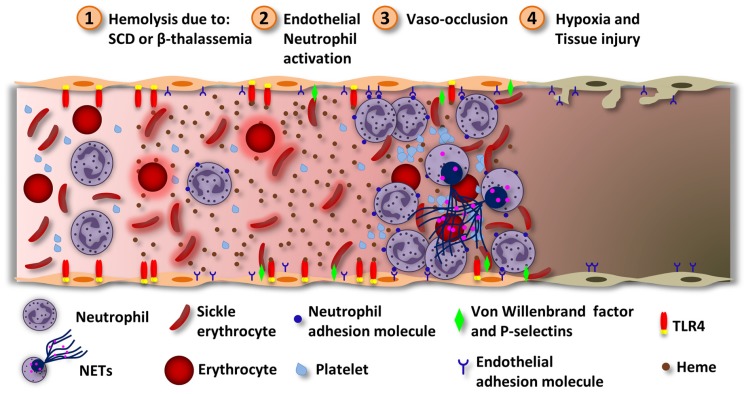
**Heme induces vascular inflammation in hemolytic diseases.** During intravascular hemolysis the serum proteins responsible for removing heme get saturated and heme can exert its inflammatory effects. (1) Hemolysis can happen due to ischemia/reperfusion, SCD or β-thalassemia. While in SCD, Hb polymerization alters erythrocytes physically, in β-thalassemia the accumulation of α-globin aggregates inside the erythrocytes. In both cases, the erythrocytes became more susceptible to hemolysis. Hemolysis increases the concentration of Hb which, under oxidative stress, releases free heme. (2) Heme activates neutrophils and endothelial cells, by ROS generation. In both cells, heme induces the expression of complementary adhesion molecules. In endothelial cells, heme induces TLR4-dependent degranulation of Weibel–Palade bodies and P-selectins and VWF release. The VWF is prothrombotic and can increase the adhesion of erythrocytes to the endothelium. Heme also induces the expression of the adhesion molecules ICAM-1, VCAM-1, and E-selectins. Neutrophils stimulated by heme and TNF releases NETs. (3) These events lead to the adhesion of neutrophils to endothelial cells. The activated endothelium now expresses molecules that can interact with surface molecules localized in erythrocytes. Also, erythrocytes express molecules that can interact with platelets. Together, these events lead to vaso-occlusion. (4) Depending on the extension of the vaso-occlusion, some tissues may experience hypoxia and damage. Vaso-occlusion of the lung microvasculature may result in the development of the ACS through the infarction of the lung parenchyma. Heme-induced TLR4 activation in endothelial cells and NETs release contribute to the ACS.

Pathologies associated with intravascular hemolysis are the most commonly related to heme inflammatory effects. SCD and β-thalassemia are molecular blood disorders caused by mutations in genes encoding Hb (Heinle and Read, 1948; Pauling et al., 1949; Ingram, 1957). In SCD, a single point mutation in the Hb gene encodes a protein that polymerizes under low-oxygen conditions causing red blood cells (RBC) deformation (sickle shape; Edelstein et al., 1973; Browne et al., 1998). Repeated sickling episodes decrease RBC elasticity rendering them susceptible to hemolysis (Hebbel, 1991). β-Thalassemia is the result of a mutation in the β-globin chain gene that impairs the β-globin chain production and leads to accumulation of the α-globin which can aggregate causing hemolysis and erythroid precursor premature death (Khandros et al., 2012). Chronic episodes of hemolyses increase the concentrations of Hb and heme which are considered to be the major responsible for vascular inflammation in these diseases (Muller-Eberhard et al., 1968; Chies and Nardi, 2001; **Figure 4**). In fact, hematin injection in healthy volunteers induces thrombophlebitis and disturbed homeostasis (Simionatto et al., 1988). Moreover, polymorphisms in the promoter region of the *Hmox1* gene is associated with decreased rates of hospitalization of patients with acute chest syndrome (ACS; Bean et al., 2012), a major life-threatening condition for patients with SCD (Gladwin and Vichinsky, 2008). Repeated vaso-occlusion episodes are characteristic of SCD and can lead to tissue damage due to IR (Kaul and Hebbel, 2000; **Figure 4**). Heme induces the expression of adhesion molecules in the vasculature (Wagener et al., 1997, 2001a; Belcher et al., 2014). The adherence of leukocytes and reticulocytes to the endothelium causes stasis and painful crisis, a hallmark of SCD pathogenesis (Belcher et al., 2000). β-thalassemic patients also experience signs of vascular inflammation and vaso-occlusion due to endothelial adhesion of aggregated thalassemic erythrocytes and reduced NO bioavailability (Bunyaratvej et al., 1995; Hovav et al., 1999; Hahalis et al., 2008). In this disease, cardiac complications seem to be the main cause of mortality (Engle et al., 1964; Spirito and Maron, 1990).

Transgenic SCD mice present typical signs of multiorgan pathology and vascular inflammation such as oxidative stress, endothelial activation, and reduced NO bioavailability (Pászty et al., 1997; Ryan et al., 1997; Belcher et al., 2003). Also, β-thalassemic mice present the same signs of vasculopathy (Vinchi et al., 2013). This phenotype is similar to that of *Hx*^-/-^ mice during hemolysis or heme overload (Tolosano et al., 1999; Vinchi et al., 2008). In both models, vascular inflammation was associated with an increase is serum heme concentration and Hx depletion. This fact is also observed in patients with hemolytic anemia (Muller-Eberhard et al., 1968; Adisa et al., 2013). Hx administration protects transgenic SCD mice and β-thalassemic mice from heme-induced oxidative stress and vascular inflammation (Vinchi et al., 2013). Hx works by chelating and delivering heme to the liver where it is catabolized by HO-1. In this context, Hx-heme complex-induced HO-1 protects the liver against heme cytotoxic effects (Vinchi et al., 2013). The increased HO-1 expression reduces vascular inflammation, vaso-occlusion and liver damage (Belcher et al., 2000; Vinchi et al., 2008, 2013). While Hx treatment protects SCD mice with severe respiratory symptoms from acute lung inflammation (ALI; Ghosh et al., 2013) and SCD mice from stasis (Belcher et al., 2014), Hx also improves cardiovascular functions in β-thalassemic mice (Vinchi et al., 2013).

Recent studies demonstrated that heme-induced endothelial cell activation is at least in part mediated by TLR4 signaling. Heme-induced TLR4 activation in endothelial cells leads to NF-κB activation, adhesion molecules expression and Weibel–Palade body degranulation (Belcher et al., 2014), which contains the VWF and P-selectins, molecules involved in SCD vasculopathy (Matsui et al., 2001; Chen et al., 2011a; **Figure 4**). Blockade of adhesion molecules, including VWF and P-selectin, inhibits heme-induced stasis in SCD mice (Belcher et al., 2014). TLR4 is also involved in heme-induced ACS. The TLR4 specific antagonist, TAK-242, which inhibits TLR4 signaling by the intracellular domain, protects transgenic SCD mice from heme-induced stasis and ACS (Ghosh et al., 2013; Belcher et al., 2014). In fact, *Tlr4*^-/-^ mice or TAK-242 administration prevented ALI and respiratory distress induced by heme (Ghosh et al., 2013). These results suggest that TLR4 could be a new therapeutic target for treating SCD.

Furthermore, antioxidants proved to be efficient modulators of vascular functions in hemolytic diseases. In fact, PKC inhibition and antioxidants also protects transgenic SCD mice from heme-induced stasis (Belcher et al., 2014). PKC was shown to mediate heme-induced ROS generation in macrophages by the Syk pathway and in neutrophils by a signaling pathway characteristic of a GPCR (Porto et al., 2007; Fernandez et al., 2010). Because ROS generation induced by heme is TLR4-independent (Figueiredo et al., 2007), it is possible that another receptor might collaborate with TLR4 to promote vascular inflammation.

Another possible mechanism involved in the pathogenesis of SCD is the release of NETs by neutrophils (Chen et al., 2014). NETs are fibers composed of chromatin (DNA and histones) decorated with antimicrobial peptides. In contrast to its functions in host defense against pathogens, which NET exert by trapping and killing them, exaggerated NET formation is implicated in a number of pathologies (reviewed by Zawrotniak and Rapala-Kozik, 2013). In this context, NET formation has been shown to contribute to disseminated intravascular coagulation in the course of inflammatory diseases and therefore to morbidity and mortality in sepsis (Fuchs et al., 2010; Massberg et al., 2010; von Bruhl et al., 2012). Thus, this effect could contribute to vaso-occlusion and ACS in the course of SCD. In fact, patients with SCD presented NET formation in plasma during steady state conditions, when compared to healthy individuals (Schimmel et al., 2013). The amount of NET formation was increased during painful crisis and ACS, and correlated with the length of hospitalization. Moreover, TNF administration in SCD mice induced NET formation within the pulmonary microvasculature (Chen et al., 2014). DNAse I treatment prevented lung inflammation and vascular permeability induced by TNF in SCD mice. In this study heme was reported to mediate NET formation in SCD mice. In vitro, TNF-primed neutrophils released NETs after the stimulation with heme. Furthermore, Hx treatment prevented TNF-induced NET formation and hypothermia in SCD mice demonstrating an important role for heme in the induction of NET formation in this model. Together, these data suggest that heme-induced NET formation might contribute to the pathogenesis of SCD (**Figure 4**).

### ATYPICAL UREMIC HEMOLYTIC SYNDROME

Atypical hemolytic uremic syndrome (aHUS) is characterized by an over activation of the complement alternative pathway (CAP) due to genetic and acquired abnormalities (Noris and Remuzzi, 2009). The pathogenesis of this disease involves glomerular endothelial damage, leukocyte activation, thrombotic microangiopathy (blood clots in small blood vessels), and mechanical hemolysis. However, the observed mutations are insufficient to induce complement abnormal activation requiring a triggering stimulus for disease manifestation. A broad range of precipitating events is related with aHUS including infections, drugs, autoimmune conditions, transplants, pregnancy, and metabolic conditions (reviewed in Loirat and Fremeaux-Bacchi, 2011). It was suggested that heme derived from hemolysis could act as a secondary hit capable of amplifying aHUS pathogenesis (deCiutiis et al., 1978; Mold et al., 1995; Ruiz-Torres et al., 2005; Pawluczkowycz et al., 2007). In fact, heme activates the CAP in the serum and on endothelial cells surfaces (Frimat et al., 2013). This effect is enhanced by mutations associated to CAP overactivation during aHUS. Heme-induced exocytosis of Weibel–Palade bodies from endothelial cells induces the expression of P-selectins in the plasma membrane, which are known to bind the complement C3b protein and trigger CAP activation, and to release the prothrombotic VWF (Frimat et al., 2013). Moreover, heme induces the release of C5a and C5b9, fragments of the C5 complement protein known to induce endothelial cell activation and permeabilization. Thus the combined effect of heme-induced CAP and TLR4 activation in endothelial cells (Frimat et al., 2013; Belcher et al., 2014) may act together to induce Weibel–Palade body degranulation, contributing to renal damage in aHUS.

### INTRACEREBRAL HEMORRHAGE

Intracerebral hemorrhage is a bleeding into the brain parenchyma. Although the mass effect of an edema derived from an expanding hematoma mediates part of the brain injury, there are evidences pointing inflammation and cytotoxic degradation products of blood as mediators of ICH injury (Xi et al., 2006). While Hx injection protects brain tissues from rat cerebral IR (Dong et al., 2013), *Hx*^-/-^ mice are susceptible to ICH (Chen et al., 2011b) suggesting a role for free heme in this process. In fact, bleeding will result in hemolysis and free heme release. Depending on experimental conditions, HO-1 can be deleterious or beneficial in neurodegenerative models. Interestingly, in a mouse model of ICH, *Hmox*^-/-^ mice present reduced brain damage (Wang and Doré, 2007) suggesting that cerebral cells are prone to the oxidative effects of free Fe derived from heme catabolism. In fact, the treatment with the Fe chelator deferoxamine ameliorates brain injury after ICH or subarachnoid hemorrhage (Gu et al., 2009; Lee et al., 2010; Chen et al., 2011c).

TLR4 has been involved in brain injury during ICH (Teng et al., 2009; Sansing et al., 2011; Fang et al., 2013; Wang et al., 2013). Exogenous heme induces brain injury through TLR4-induced inflammation (Lin et al., 2012). In fact, *Tlr4*^-/-^ or anti-TLR4 treatment suppresses heme-induced neuroinflammation, edema, and neurologic deficit. Moreover, heme activates microglia cells to produce TNF, IL-6, and IL-1β through TLR4 signaling indicating that heme can directly activate brain cells (Lin et al., 2012). Heme-induced IL-1β production during neuroinflammation suggests the involvement of the NLRP3 inflammasome. In macrophages, heme induces IL-1β secretion via mROS generation and NLRP3 activation (Li et al., 2014; unpublished data). Interestingly, NLRP3 knockdown and mROS inhibitors reduce brain edema and improve neurological functions (Ma et al., 2014).

### ATHEROGENESIS

Atherosclerosis is a chronic inflammatory disease characterized by the formation of plaques in the vessel walls (Libby et al., 2002). Plaque formation is initiated by lipid accumulation in subendothelial spaces and metabolic dysfunction in infiltrating monocyte-derived macrophages which contributes to foam cells development (Moore and Tabas, 2011). Part of these cells die and release their fat and cholesterol-laden membranes into the intercellular space recruiting more monocytes in a chronic process that increases the formation of the necrotic core (Guyton and Klemp, 1996; Virmani et al., 2005). The necrotic core rupture can lead to serious complications. The release of plaque components into the blood stream form clots that damages the heart muscle and slowly develop ischemic heart disease (Falk et al., 1995; Arroyo and Lee, 1999). Also, if these clots are big enough they can block blood flow and induce tissue necrosis. On the other hand, the necrotic core rupture exposes inflammatory components which can bind platelet cells at the rupture site and induce their accumulation forming thrombus which can be large enough to produce vessel lumen narrowing (Virmani et al., 2005).

Low-density lipoprotein is the major lipid involved in plaque formation. In the inflammatory environment, subendothelial LDL is oxidized and becomes a potent inflammatory molecule. oxLDL activates intraplaque macrophages by the TLR4–TLR6–CD36 complex engagement leading to the activation of the NFκB pro-inflammatory pathway and facilitating endocytosis (Stewart et al., 2010). oxLDL endocytosis leads to the formation of crystalline substances inside lysosomes causing the destabilization of this organelle as well the release of proteases, events that lead to the NLRP3 inflammasome activation (Duewell et al., 2010). Indeed, TLRs and NLRP3 have been associated with atherosclerosis development.

Low-density lipoprotein oxidation and its inflammatory effects can be enhanced by the development of new vessels inside the necrotic core (Virmani et al., 2005). The major stimulus for this angiogenic process seems to be hypoxia (Bjornheden et al., 1999; Sluimer et al., 2008). In fact, the plaque atheroma composition decreases oxygen availability rendering the necrotic core structure a hypoxic condition. Furthermore, inflammatory cells can actively contribute to angiogenesis by secretion of angiogenic factors, such as vascular endothelial growth factor (VEGF), fibroblast growth factor 2 (FGF2), and platelet-derived growth factor BB (PDGFBB) ligands (Cheng et al., 2013). The new vessels generally arise from vasa vasorum (Kwon et al., 1998), a network of small blood vessels that supply the walls of large blood vessels. Plaque new vessels are immature and present a fragile structure which is prone to leakage and highly vulnerable to rupture, thus causing intraplaque hemorrhage (Kolodgie et al., 2003; Dunmore et al., 2007; Sluimer et al., 2009). This event is responsible for increased entry of erythrocytes and inflammatory cells inside the necrotic core. Erythrocytes that enter developing atherosclerosis lesions are prone to lysis by lipid oxidation products. Erythrocytes lysis increases the amount of extracellular Hb which in a highly oxidative environment leads to MetHb formation and the release of free heme. LDL oxidation induced by heme increases the oxidation of lipids and inflammatory stimuli, contributing to further plaque development (Balla et al., 1991a). In fact, heme-induced LDL oxidation is highly cytotoxic for endothelial cells and LDL oxidation seems to be mediated by Fe (Jeney et al., 2002; Nagy et al., 2010). This group of events, triggered by plaque hemorrhage, increases plaque vulnerability and consequently raises the risk of thrombus formation and ischemic heart disease (Michel et al., 2011). Expression of HO-1 provides protection against several cardiovascular diseases, including atherosclerosis (reviewed by Wu et al., 2011). Interestingly, a 6-year-old patient with HO-1 deficiency experienced a severe atherosclerotic pathology (Yachie et al., 1999). This patient suffered from persistent intravascular hemolysis, anemia, endothelial damage, iron deposition, and cellular sensibility to heme cytotoxic effects. These observations resemble those of *Hmox*^-/-^ mice, thus demonstrating the importance of HO-1 functions in the maintenance of iron recycling and tissue homeostasis.

After hemolysis, sustained interaction between Hb and ROS can lead to ferrylhemoglobin (ferrylHb) formation, which is characterized by an increase in the Fe oxidative state to Fe^+^^4^ (Harel and Kanner, 1988; Patel et al., 1996). ferrylHb undergoes intermolecular cross-linking of its globin chains forming aggregates, which induces the expression of adhesion molecules in vascular endothelial cells that support the recruitment of macrophages into the vessel wall (Silva et al., 2009; Potor et al., 2013). Similarly to heme, ferrylHb activates endothelial cells through NFκB activation (Silva et al., 2009). However, the mechanism responsible for NFκB activation is different. While TLR4 and ROS mediate heme-induced activation of macrophages and endothelial cells (Figueiredo et al., 2007; Belcher et al., 2014), ferrylHb activates endothelial cells independently of TLR4 and ROS (Silva et al., 2009). Moreover, ferrylHb is unable to induce cytokine secretion by endothelial cells (Silva et al., 2009), another difference to heme which induces IL-8 production (Natarajan et al., 2007). Like MetHb, ferrylHb is unstable and releases free heme to further increase oxLDL formation (Potor et al., 2013). Thus, heme and ferrylHb can induce direct effects, such as endothelial cells activation, and indirect effects, like LDL oxidation to increase atheroma plaque development and atherosclerosis pathogenesis.

Besides heme pro-inflammatory capacity in atherosclerotic plaque development, it was proposed that heme could mediate an atheroprotective event. Recently, heme was shown to activate human monocytes by the induction of activating transcription factor 1 (ATF-1), coupling the expression of HO-1 and liver X receptor (LXR)-β to induce a specific macrophage phenotype (Boyle et al., 2012). HO-1 is well known for its homeostatic functions creating an antioxidant and cytoprotective environment against heme and Fe deleterious effects (Gozzelino et al., 2010). LXR-β protects against lipid overload by activating a lipid exportation program regulated by proteins such as LXR-α and ATP binding cassette transporter A1 (ABCA1), preventing foam cells formation. This phenotype induced by heme is distinct from M1, M2, and Mox (reviewed in Moore et al., 2013) and therefore was named Mhem. Mhem was described in macrophages from atheroma plaques of patients with fatal coronary artery disease (Boyle et al., 2009). In this context, ATF-1 is able to convert macrophages to an atheroprotective state coordinating Fe and lipid metabolism.

The Mhem phenotype contrasts with the Fe-loaded M1 macrophages phenotype which presents an enhanced production of TNF and hydroxyl radicals, and has the capacity to induce precocious fibroblast senescence, impairing wound healing (Sindrilaru et al., 2011). Macrophages with this phenotype were described in the skin of patients with chronic venous leg ulcers (CVUs). This disease is caused by chronic venous valve insufficiency that leads to hypertension in the lower-limb veins, with persistent erythrocyte extravasation. Erythrophagocytosis by tissue macrophages and release of heme-bound Fe seems to be the trigger of this pro-inflammatory phenotype. It will be interesting to investigate the presence of Mhem in other pathologies such CVUs to understand the environments and factors involved in the development of Mhem and its capacity to modulate pro-inflammatory microenvironments also induced by heme and Fe. Moreover, it will be interesting to characterize the putative involvement of innate immune receptors activated by heme in these processes.

### HEMOLYSIS DUE TO INFECTION

Vascular hemolysis is a relevant factor in some infectious diseases. The severe pathology observed in malaria and hemorrhagic fevers is a result of complex events that triggers a vigorous inflammatory response. The systemic inflammatory response and the disseminated intravascular coagulation observed in severe cases of these diseases are similar to septic shock and are also dependent on the production of high amounts of inflammatory mediators such as TNF, IL-6, and IL-1β. Heme homeostasis is critically involved in the development of sepsis and malaria (Pamplona et al., 2007; Larsen et al., 2010; Gozzelino et al., 2012). In both cases it is possible to observe high amounts of heme in the circulation (**Figure 4**). In malaria, it was demonstrated that heme acts together with high amounts of ROS to induce the disruption of the blood–brain barrier permitting the migration of inflammatory cells, plasmatic proteins, and *Plasmodium* antigens, which cause damage to brain tissues (Pamplona et al., 2007). In the CLP (cecal ligation and puncture) model of sepsis, heme sensitizes hepatocytes to necrotic cell death rendering mice more susceptible to sepsis (Larsen et al., 2010). Moreover, there is a negative correlation between Hx serum concentration and tissue damage in patients with septic shock. Therefore, patients with septic shock presenting higher serum concentrations of Hx develop decreased tissue damage and have a better survival outcome, suggesting an important role for heme during sepsis (Larsen et al., 2010). As discussed, Hx inhibits several pro-inflammatory effects induced by heme such as ROS generation, cell death (Larsen et al., 2010; Gozzelino et al., 2012), and the synergistic effect (Lin et al., 2010) with PAMPs. HO-1 has a central role in maintaining tissues homeostasis against heme noxious effects, since *Hmox*^-/-^ mice are extremely susceptible to CLP and cerebral malaria (Pamplona et al., 2007; Larsen et al., 2010). In both cases, HO-1 maintains tissue homeostasis independently of parasite burden. Thus, HO-1 confers tolerance against these infectious diseases. On the other hand, HO-1 induction confers host resistance, rather than tolerance, to *Mycobacterium* infection (Silva-Gomes et al., 2013). *Hmox1-/-* mice infected with *M. avium* presented a susceptible phenotype with an increased pathogen burden due to impaired protective granuloma formation. HO-1 deficiency induced heme accumulation and macrophage cell death which contributed to *M. avium* proliferation. In fact, heme administration to macrophages in vitro increased *M. avium* proliferation and heme injection in infected mice prevented granuloma formation. Moreover, *Hmox1-/-* mice infected with M. tuberculosis died while WT and heterozygous mice (*Hmox1+/-*) survived. Thus, HO-1 plays a critical role during *Mycobacterium* infection by preventing heme-induced granuloma macrophage death and bacterial proliferation. Besides its role in tissue tolerance and bacterial control, HO-1 antioxidant activity might contribute to intracellular bacterial multiplication. In fact, HO-1 impairs resistance to *Plasmodium* infection in the liver (Epiphanio et al., 2008) and to non-typhoid *Salmonella* (NTS), a common complication of *P. falciparum* infection (Cunnington et al., 2012a, b). In fact, hemolysis triggered by *Plasmodium* infection causes premature mobilization of bone-marrow granulocytes with impaired antioxidant defenses due to HO-1 expression (Cunnington et al., 2012a, b). This leads to uncontrolled bacterial load and lethal bacteremia. Together these observations reinforce the concept that heme might be used as a target for adjuvant therapies during infectious diseases.

## FUTURE PERSPECTIVES

The last 10 years of research on heme inflammatory properties brought new insights to understand its functions in hemolytic and hemorrhagic pathologies. Besides the fact that heme presents direct cytotoxic properties, it is now clear that heme can activate specific receptors and signaling pathways to promote ROS generation, inflammation, and programed cell death. Important studies demonstrated that heme-induced TLR4 activation is involved in the pathogenesis of SCD and ICH. Interestingly, a specific inhibitor of TLR4 signaling is very efficient preventing heme-induced vasculopathy and lung injury in mouse models of these diseases. Moreover, heme activates the NLRP3 inflammasome inducing IL-1β processing and secretion. The critical role of the inflammasome in the pathological response following lethal hemolysis was unveiled. Moreover, new studies are helping to understand the beneficial properties of heme scavenging proteins such as Hx. In fact, Hx was shown to protect mice against infectious (malaria and sepsis) and non-infectious (SCD, β-thalassemia, and cerebral IR) models of diseases. Characterizing the role of cell death pathways induced by heme and the participation of heme transporters on hemolytic diseases promises interesting perspectives. Thus, understanding the molecular signaling pathways affected by heme might prove useful to the identification of new options for treating pathological conditions that course with increased extracellular heme and inflammation.

## Conflict of Interest Statement

The authors declare that the research was conducted in the absence of any commercial or financial relationships that could be construed as a potential conflict of interest.
